# Recent Advances in Fluorinated Colloidal Nanosystems for Biological Detection and Surface Coating

**DOI:** 10.3390/polym18030316

**Published:** 2026-01-24

**Authors:** Fei Xu, Xiaolong Cao, Kai Yan

**Affiliations:** 1 Jiaxing Key Laboratory of Preparation and Application of Advanced Materials for Energy Conservation and Emission Reduction, College of Advanced Materials Engineering, Jiaxing Nanhu University, Jiaxing 314001, China; 2 College of Bioresources Chemical and Materials Engineering, Shaanxi University of Science & Technology, Xi’an 710021, China

**Keywords:** fluorinated colloidal nanosystems, biological detection, surface coating

## Abstract

Fluorinated colloidal nanosystems have attracted significant attention for their advantageous properties and potential applications in the biomedical field, especially in ^19^F magnetic resonance imaging. These nanosystems are known for their high specificity, excellent biocompatibility, and ease of functional modification. Furthermore, they offer unique advantages for functional surface coating due to their surface performance and chemical resistance. This paper discusses recent developments in fluorinated colloidal nanosystems, including applications in biological detection (such as enzymes, proteins, pH levels, ions, reducing environments, and reactive oxygen species) and surface coating (such as self-cleaning, self-healing, antibacterial properties, anti-fogging, antifouling, and oil–water separation). This article also highlights current challenges and provides suggestions for future research directions in the field of fluorinated colloidal nanosystems.

## 1. Introduction

Significant interdisciplinary research has been focused on fluorinated colloidal nanosystems for energy storage and conversion [1] and environmental and biological applications [2,3]. These nanosystems are valued for their exceptional chemical resistance, biocompatibility, thermal stability, low surface energy, minimal friction coefficients, and electrical properties. The advancements in clinical biomedicine, nanotechnology, and materials science have spurred the development of diverse fluorinated colloidal nanosystems [4,5], showcasing their utility in biological detection and surface coating.

Magnetic resonance imaging (MRI) is based on the principle of nuclear magnetic resonance (NMR). According to the different arrangements and precession of spin nuclei in different structural environments inside the material in the external gradient magnetic field, the structure image inside the object is drawn. Currently, hydrogen spectrum-based MRI has been used for clinical monitoring. However, MRI based on the hydrogen spectrum is prone to produce strong background interference, which affects the diagnostic results of magnetic resonance imaging. Therefore, magnetic resonance imaging technology based on heteronuclear molecules (such as ^13^C, ^23^Na, ^31^P or ^19^F) has received widespread attention. The ^19^F atom has excellent nuclear magnetic resonance properties: 100% natural abundance, spin quantum number is 1/2, a comparable gyromagnetic ratio with the ^1^H nucleus (40.08 vs. 42.58 MHz/T), and sensitivity is 83% of the proton. In addition, the ^19^F atom is more sensitive to the local environment, and the chemical shift range is wider (>350 ppm). And only trace amounts (<10^−6M^) of ^19^F exist in bones and teeth in solid form, and the background signal is much lower than the detection limit of MRI [6]. Therefore, the ^19^F atom becomes another imaging atom after ^1^H MRI and plays a crucial role in ^19^F MRI. This imaging technique not only guides drug delivery and monitors treatment but also enables personalized administration [7]. Particularly interesting are smart or activatable ^19^F probes for detection, allowing real-time acquisition of dynamic processes and functional information for more specific diagnoses [8]. Additionally, the fluorine atom has the characteristics of large electronegativity, a small radius, and a low polarization rate, so the bond energy of forming a carbon–fluorine covalent bond is very large, which can effectively reduce the surface tension of water. At the same time, fluorine atoms can protect carbon atoms and carbon chains, making it difficult for other atoms to wedge, so that the fluorinated polymers (polymers containing C–F bonds) show the characteristics of more stability, acid and alkali resistance, and high temperature resistance. Fluorocarbon polymer materials prepared by solution polymerization or emulsion polymerization using fluorine-containing polymer materials and other monomers have been well used in the modern aerospace, military, and agriculture industries because of their high temperature resistance, superior weather resistance, excellent self-cleaning performance, and corrosion resistance. To sum up, the cutting-edge research on emerging fluorinated multifunctional nanosystems is garnering increasing attention in the scientific community, particularly in the fields of biomaterials and surface coatings.

**Figure 1:**
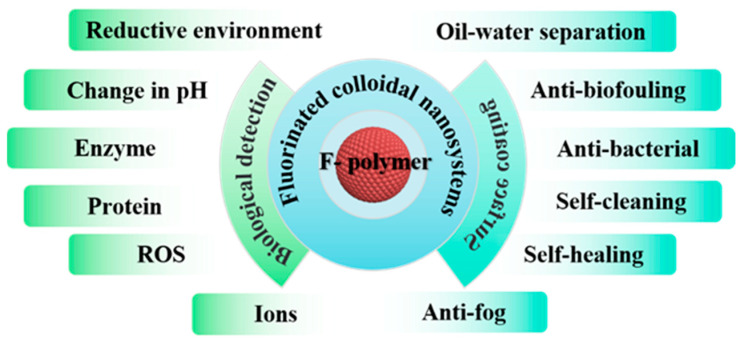
Schematic illustration of fluorinated colloidal nanosystems.

This review summarizes the recent advancements in fluorinated colloidal systems, focusing on their applications in biological detection and surface coating (Figure 1). Specifically, it discusses the current utilization of ^19^F MRI in detecting various biological entities like pH, enzymes, proteins, ions, reactive oxygen species (ROS), and reductive environments. Furthermore, it introduces the application of fluorinated components in functional surface coatings for self-cleaning, self-healing, antibacterial, anti-fog, antifouling, and oil–water separation purposes. The review concludes by outlining future research directions and potential applications of fluorinated polymers.

## 2. ^19^F MRI

In recent years, MRI technology has become commonly used in clinical settings for diagnosing diseases and monitoring treatment progress [9]. The key advantages of MRI, such as deep tissue penetration, minimal invasiveness, and superior spatial resolution, set it apart from other imaging techniques [10]. Despite the success of using paramagnetic relaxation agents in clinical applications (with over 10 million gadolinium-enhanced MRI procedures conducted each year), one major drawback is the lack of image clarity, leading to high background noise and difficulties in accurately measuring concentration levels in living organisms. The imaging of MRI agents containing gadolinium or iron oxide nanoparticles presents challenges as the adjacent tissues often appear dark [11]. Furthermore, iron oxide nanoparticles create negative contrast and rely on altering the ^1^H MRI signal, resulting in dark regions against a white backdrop [12]. Considering that other natural phenomena can also produce dark regions, it requires meticulous analysis of error-prone images. Gadolinium creates a favorable contrast in T_1_-weighted images; however, this contrast overlaps with the regular ^1^H image in the backdrop [13]. To address the limitations of traditional ^1^H imaging agents, a promising approach is the advancement of “second color” imaging through the use of heteronuclear MRI atoms, like ^13^C, ^23^Na, ^31^P, or ^19^F, alongside ^1^H.

Among these imaging nuclei, ^19^F seems the most beneficial due to its advantageous NMR properties and minimal biological background caused by the low levels of native fluorine in the body [14]. Additionally, ^19^F proves promising with its 100% natural abundance and a gyromagnetic ratio (40.06 MHz/T) just below that of ^1^H, offering enhanced detection sensitivity compared to other nuclei [15]. The chemical shifts of ^19^F cover a broad range (>350 ppm), making it a valuable tool for quantitative imaging applications due to the absence of endogenous ^19^F signals and the correlation between ^19^F concentration and signal intensity [16]. Magnetic resonance (MR) images rely on signals from water molecules in biological tissues, whereas ^19^F MRI specifically utilizes fluorine atoms present in fluorinated imaging agents. In spin-echo MR imaging, signal intensity within a specific volume is determined by the number of spins and their relaxation times (T_1_ and T_2_), as shown in the equation below.

$$Imaging\;Intrnsity\approx N(F)\left[1-2exp\left(\frac{-\left(T_{R}-T_{E}/2\right)}{T_{1}}\right)+exp\left(\frac{-T_{R}}{T_{1}}\right)\right]exp\left(\frac{-T_{E}}{T_{2}}\right)$$

In this equation, N(F) represents the detectable ^19^F nuclei, TR and TE are the repetition time and echo delay time in the pulse sequence, respectively. According to the equation, high sensitivity of the MRI signal requires high ^19^F content, long T_2_, short T_1_, and chemical uniformity. Therefore, ^19^F MRI contrast agents should have biological stability, extended shelf life, minimal toxicity, and a distinct, strong, and singular ^19^F NMR peak in vitro and in vivo. Researchers are focusing on developing new ^19^F MRI CAs to meet these criteria. According to our previous review paper [17], ^19^F MRI CAs are classified into two main types: perfluorocarbons-based emulsion, where perfluorocarbons encapsulated in a polymer emulsion act as the ^19^F source, and partly fluorinated polymers, including linear, (hyper)branched, and dendritic polymers.

## 3. Detection of Biological Activity

Chemical tools for precise sensing and identification of biological activities play a crucial role in basic medical and biological investigations [18]. Among various methods of detection, ^19^F NMR/MRI is expected to serve as a valuable tool for deep tissue and noninvasive detection. Currently, numerous ^19^F probes with targeting and control properties are actively being developed [19]. Targeting probes are expected to accumulate in specific environments by binding to local components, resulting in changes in ^19^F chemical shifts. On the other hand, switching probes exhibit activation based on paramagnetic relaxation enhancement during cleavage reactions. The majority of probe signals are weakened as the molecules aggregate, but they regain strength upon disassembly.

### 3.1. Enzyme Detection

**Figure 2:**
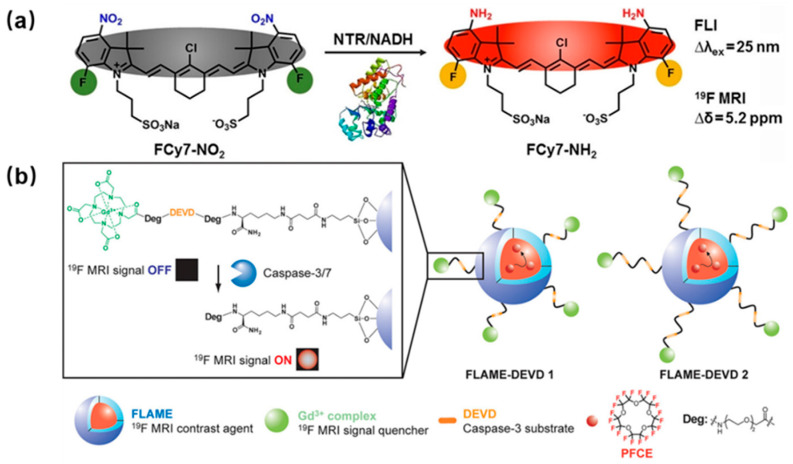
(**a**) Schematic diagram of the probe FCy7-NO2 generating the target probe FCy7-NH2 under the action of nitroreductase. Reprinted from Ref. [20] with permission from Wiley. (**b**) Schematic diagram of enzyme-responsive 19F MRI nanoprobes for detecting caspase-3/7 activity. Reprinted from Ref. [22] with permission from American Chemical Society.

Detection of enzyme levels and activity holds significant importance in studies related to drug development and medical diagnostics. Chen et al. designed a dual-mode near-infrared (NIR) fluorescence and ^19^F MRI probe FCy7-NO_2_ for nitroreductase imaging (Figure 2a) [20]. This probe combines fluorine and nitro groups with Cy7, and upon retrobulbar intravenous injection, the enzyme nitroreductase triggers the formation of the target probe FCy7-NH_2_ in the area of lung cancer. This enzymatic reaction can be detected using both fluorescence and ^19^F MRI techniques due to the low fluorescence excitation wavelength of FCy7-NH_2_. The findings indicate that the combination of ^19^F NMR and fluorescence enables the quantification of nitroreductase levels across a broad range, outperforming individual imaging methods and providing clinicians with an effective tool for disease assessment. Buzhor et al. developed amphiphilic hybrids with a modular structure comprising a hydrophilic polyethylene glycol block and a hydrophobic enzyme-responsive 4-(trifluoromethyl) benzene labeling group [21]. These hybrids self-assemble into intelligent micellar nanocarriers. Upon activation by porcine liver esterase and cleavage of the fluorinated groups, the polymeric micelles disassemble, leading to the ON state of the MR signals due to increased hydrophilicity and relaxation.

Moreover, observing enzyme activities in real-time within living organisms could offer valuable insights into the understanding of various illnesses. Caspase-3 serves as a crucial enzyme in the process of apoptosis and has been extensively utilized in assessing the effectiveness of anticancer treatments that induce cell death in tumor cells. Multiple investigators have conducted evaluations on the efficacy of these agents. As depicted in Figure 2b, Akazawa et al. developed a Gd-DOTA-DEVED-T ^19^F MRI probe, comprising a Gd^3+^ complex, an enzyme substrate peptide, and a ^19^F-containing moiety [22]. Upon exposure to caspase-3 at 37 °C, the DEVED peptide sequence was cleaved, resulting in the attenuation of the intramolecular paramagnetic effect from Gd^3+^ to ^19^F. The intensity of the ^19^F NMR peak increased over time during incubation. Furthermore, the probe exhibited the ability to spatially detect caspase-3 activity from the phantom image using ^19^F MRI.

### 3.2. Detection of Protein

**Figure 3:**
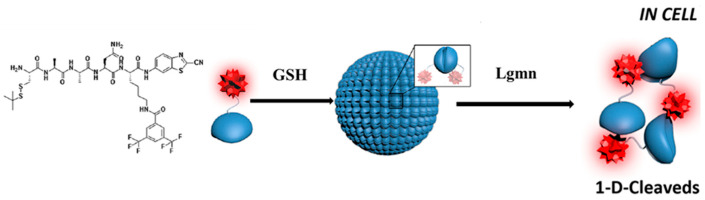
Schematic illustration of intracellular GSH-controlled self-assembly followed by Lgmn-controlled disassembly of nanoparticle. Reprinted from Ref. [25] with permission from American Chemical Society.

Proteins possess attractive biocompatibility, impressive degradability, and various functionalities. They have garnered growing interest in drug/gene delivery, protein therapy, nanoreactors, and synthetic cells [23]. As a result, the identification and recognition of proteins carry significant research importance in life sciences. A promising method for such detection is ^19^F NMR, which exhibits high sensitivity towards alterations in the microenvironment surrounding the protein. Takaoka and colleagues successfully synthesized supramolecular organic nanoparticles containing a 3,5-bis(trifluoromethyl)benzene derivative for selective protein identification employing a protein ligand-tethered self-assembling ^19^F probe [24]. Upon dissolving the nanoparticles in a buffer solution containing human carbonic anhydrase I proteins, a distinct ^19^F NMR signal emerged at −62.6 ppm. The signal strength rose steadily in correlation with the protein concentration, enabling the technique to provide quantitative data. Furthermore, Legumain (Lgmn) has been identified in leguminous plant seeds, playing vital roles in normal physiology and the prevention of inflammatory diseases. Due to its possession of distinct peptide substrates, it is feasible for chemists to devise ^19^F MRI probes for their detection. In the examination presented in Figure 3, Yuan et al. produced a clever ^19^F probe Cys(StBu)-Ala-Ala-Asn-Lys(FMBA)-CBT primarily consisting of 3,5-bis(trifluoromethyl)benzene and cysteine that forms disulfide bonds [25]. The cyclic oligomers were able to form nanoparticles through reduction by glutathione within cells, after which they would break apart in response to encountering Lgmn. This unique ability to assemble and disassemble gave the polymeric probe the ability to switch between “OFF” and “ON” states for ^19^F signals. Utilizing this responsive mechanism, the researchers effectively used the polymeric probe to detect low levels of Lgmn activity through ^19^F MRI in zebrafish under 14.1 T magnetic field strength.

### 3.3. Detection of Changes in pH

**Figure 4:**
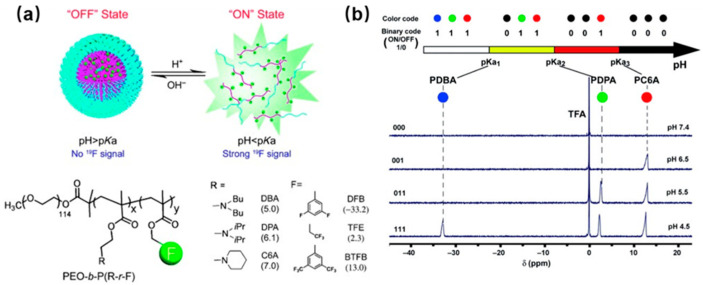
(**a**) Schematic illustration of pH-responsive nanoprobes for ^19^F-MRI with switchable ON/OFF capabilities. (**b**) A diagram depicting the activation barcode concept enables the direct determination of pH levels within neighboring pKa values. Reprinted from Ref. [28] with permission from Wiley.

pH is a critical physiological parameter present in both the intracellular and extracellular environments. An abnormal pH level is often linked to cancer, where cancer cells exhibit a unique “reverse” pH gradient across the cell membrane compared to normal cells [26]. The significance of pH values extends to guiding health and wellness. To assess its importance, Dawid et al. devised a pH-sensitive ^19^F MRI contrast agent utilizing a fluoride hydrazone molecular switch containing a dangling paramagnetic complex [27]. The switch undergoes isomerization upon changes in pH, leading to a gradual increase in T_1_ and T_2_ relaxation times in MRI. Consequently, the distance between the fluorine atom and the paramagnetic center changes, facilitating ^19^F pH imaging based on relaxation rates. Employing this probe in ^19^F MRI enables the detection of specific molecular and physiological events. In the study, various ^19^F reporters were incorporated into ionizable amphiphilic copolymers consisting of a hydrophilic poly(ethylene oxide) segment and tertiary amine/ammonium segment (Figure 4a) [28]. When the pH exceeds the pKa value, hydrophobic micelle formation occurs, restricting chain movement and causing short spin–spin relaxation times, which ultimately results in the broadening and disappearance of ^19^F signals. Conversely, at pH levels below the pKa, protonation of ammonium groups causes the micelles to disintegrate, leading to increased flexibility in the dissociated polymer chains (unimers) and the subsequent restoration of the original ^19^F signal. These nanoprobes demonstrate a high sensitivity to pH changes (pH ON/OFF = 0.25 pH unit) and can function as binary indicators for specific pH transitions (Figure 4b).

### 3.4. Detection of Ions

**Figure 5:**
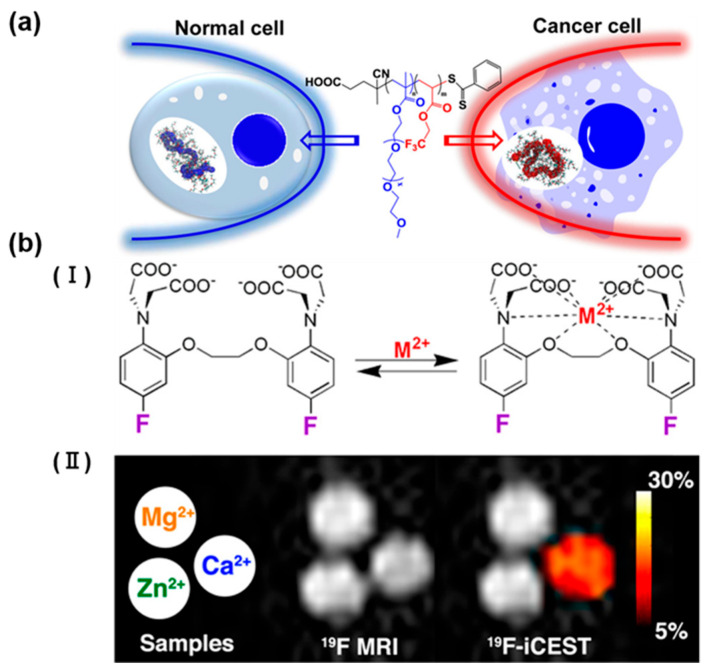
(**a**) Schematic illustration of pH-responsive nanoprobes for ^19^F-MRI with switchable ON/OFF capabilities. Reprinted from Ref. [30] with permission from American Chemical Society. (**b**) A diagram depicting the activation barcode concept enables the direct determination of pH levels within neighboring pKa values, [**I**] schematic depiction of the dynamic exchange process between free 5F-BAPTA and M^2+^-bound [M^2+^-5F-BAPTA], [**II**] ^19^F MRI and iCEST images of M^2+^ solutions. Reprinted from Ref. [31] with permission from American Chemical Society.

Metal ions play a vital role in various biological processes; thus, accurate monitoring of specific metal ions and their local concentrations using innovative imaging technology is crucial for a precise understanding of these processes [29]. It is well-known that cancerous tissue often shows higher levels of Na^+^ compared to normal tissue. Consequently, several studies have explored indicators for detecting sodium ions using ^19^F NMR. Zhang et al. developed a new ion-responsive copolymer poly(OEGA-co-TFEA) via reversible addition–fragmentation chain transfer polymerization [30]. The polymer’s conformation and mobility changes were evaluated by measuring its hydrodynamic diameter and ^19^F T_2_ relaxation time in both aqueous and salt solutions. In MCF-7 cancer cells, the recorded ^19^F NMR T_2_ value (82.3 ms) was significantly lower than in normal cells (124.2 ms), providing enough contrast for detection using advanced magnetic resonance imaging techniques in high-field scanners (Figure 5a). These findings demonstrate that the ^19^F NMR T_2_ relaxation time of fluorinated polymers can be a robust marker for identifying cancerous tissue. Additionally, chemical exchange saturation transfer introduces a new mechanism for enhancing NMR contrast, where radiofrequency-labeled protons undergo chemical exchange with bulk water protons. The substances’ information can be indirectly inferred by monitoring changes in water molecule signals. The ion-complexed sensor’s ^19^F frequency is radiofrequency-labeled, causing a significant reduction in the free sensor’s ^19^F NMR signal through magnetization transfer. This chemical exchange leads to a detection sensitivity enhancement of 10 to 1000 times. Shir et al. utilized 5,5′-difluoro derivative of 1,2-bis(oaminophenoxy) ethane-N,N,N′,N′-tetraacetic acid (5F-BAPTA) for calcium (Ca^2+^) binding (Figure 5b) [31]. They investigated the chemical shift variations and enhancement of the ^19^F signal in chemical exchange saturation transfer (CEST). A ^19^F CEST experiment was conducted to assess solutions containing Ca^2+^ (slow to intermediate exchange), Zn^2+^ (very slow exchange), and Mg^2+^ (fast exchange) using 5F-BAPTA. Notably, a significant saturation transfer contrast was identifiable solely in the Ca^2+^-rich solution. By monitoring alterations in signal intensity, they were able to indirectly identify low concentrations of Ca^2+^ with heightened sensitivity. Additionally, 5,5′,6,6′-tetrafluoro-BAPTA was employed as the ^19^F iCEST probe. Through leveraging the dynamic exchange between ion-bound and free TFBAPTA, MRI contrast was achieved using multi-ion chemical exchange saturation transfer. The study demonstrated the specific and simultaneous detection of Zn^2+^ and Fe^2+^ ions [32].

### 3.5. Reductive Environment

**Figure 6:**
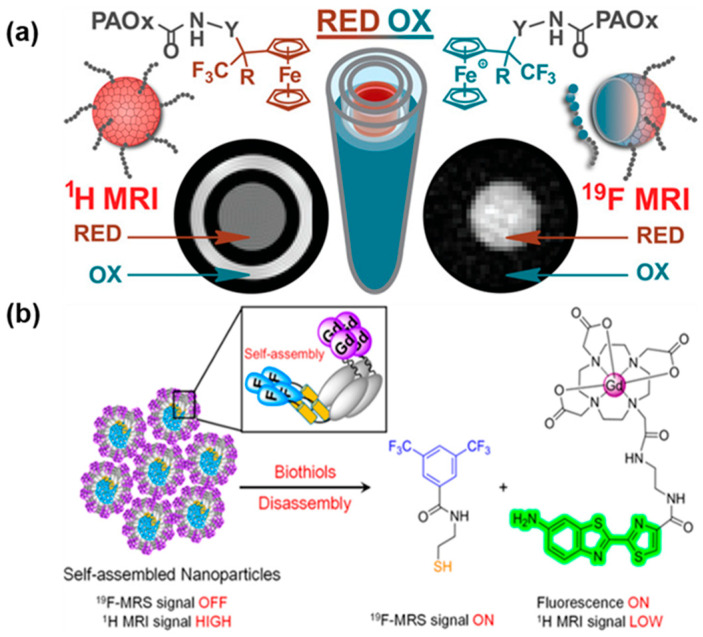
(**a**) Redox-responsive polymer with fluorinated ferrocene for ^19^F MRI theranostics. Re-printed from Ref. [34] with permission from American Chemical Society. (**b**) A triple-functional probe of fluorescence/^19^F-MRS/^1^H-MRI upon redox activation. Reprinted from Ref. [35] with permission from American Chemical Society.

In comparison to the regular physiological conditions, the pathological condition often displayed symptoms such as acidosis, lack of oxygen, increased expression of specific proteins, and an imbalance in redox. In response to biological stimuli, researchers have extensively studied and utilized activatable probes for imaging and drug delivery to enhance diagnostics and treatment, and the outcomes were focused on targeted delivery, triggering activation, and managing release [33]. Pavel Švec and colleagues developed an amphiphilic redox-responsive poly(2-oxazoline)s bearing fluorinated ferrocene moieties [34]. In a redox state, the conversion of non-magnetic fluorinated ferrocenes to magnetic ferrocenes occurs owing to the partial oxidation of the ferrocenes (Figure 6a), leading to a significant change in the chemical shift and relaxation time of the ^19^F nucleus. This amphiphilic polymer showed promising application for selectively imaging ROS-related processes and drug delivery in vivo.

Abnormal redox status has been linked to various health conditions, including cancer, liver damage, and Alzheimer’s disease. Different probes are used to detect changes in the cellular environment. Zheng et al. developed a triple-functional probe (Figure 6b) that can detect redox changes [35]. This probe combines a Gd^3+^ chelate linked to a quenched amino oxyluciferin fluorophore and a (3,5-bis(trifluoromethyl)benzene moiety connected to the amino oxyluciferin via a disulfide linker. The molecular probe can form nanoparticles with suppressed fluorescence, weakened ^19^F-MRS signal, and enhanced ^1^H-MRI contrast. When the disulfide linker is cleaved in a reducing environment, the probe disassembles rapidly, releasing a ^19^F moiety and a fluorescent Gd^3+^-chelate. These results show significant improvements such as a 70-fold increase in fluorescence activation, a 30-fold amplification of ^19^F-MR signal, and a 68% decrease in r_1_ relaxivity at 0.5 T. Hence, this functional probe has been successful in detecting reductive biothiol species. Employing a similar disulfide bond cleavage mechanism, Nakamura et al. utilized the disulfide linker to detach Gd complexes from silica nanoparticles and subsequently loaded perfluorocrown-5-ether (PFCE) [36]. Despite the PFCE core of the nanoparticle being approximately 250 Å away from the Gd^3+^ complexes on the surface, the paramagnetic relaxation enhancement effect effectively reduces the ^19^F NMR/MRI signals. Upon exposure to a reducing agent like tris(2-carboxyethyl)-phosphine, the chelated Gd^3+^ is detached from the nanoparticle surface, activating the ^19^F NMR signals of PFCE.

### 3.6. ROS Detection

**Figure 7:**
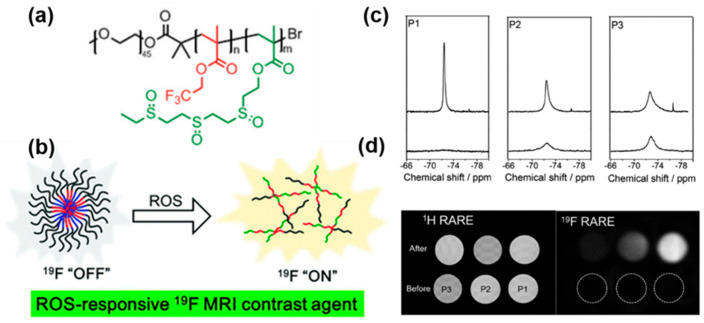
(**a**) Molecular diagram of PEG-b-poly (TFEMA-co-ETEMA) copolymers. (**b**) ROS-responsive diagram of ^19^F MRI contrast agent. (**c**) ^19^F NMR graphs of three polymers pre- (bottom) and post-oxidation (top). (**d**) ^1^H MRI and ^19^F MRI fake images of three polymers pre- and post-oxidation. Reprinted from Ref. [39] with permission from Royal Society of Chemistry.

Reactive oxygen species (ROS) are a group of chemically active molecules or ions exhibiting high oxidation activity. This category comprises hydrogen peroxide (H_2_O_2_), hydroxyl radical (OH), peroxynitrate (ONOO^−^), superoxide (O_2_^−^), hypochlorous acid (HClO), singlet oxygen, and other species [37]. Typically, reactive oxygen species are generated from mitochondrial electron transport. While appropriate levels of ROS play a crucial role in signal transduction within healthy cells, excessive ROS can harm various biological macromolecules, such as lipids, nucleic acids, and proteins, leading to disruptions in their normal physiological functions. Numerous research studies have established a direct correlation between ROS and various pathological conditions like cancer, atherosclerosis, diabetes, and inflammation [38]. The employment of MRI techniques for ROS visualization represents a valuable asset for the identification and subsequent management of such diseases. Fu and colleagues developed imaging agents using thioether- and fluorine-containing methacrylate monomers through atom transfer radical polymerization [39]. The movement of fluorinated segments within the structures is restricted, resulting in decreased ^19^F T_2_ relaxation times and a decrease in the ^19^F NMR signal intensity (Figure 7a,b). Upon exposure to ROS (H_2_O_2_), the hydrophobic thioether groups of the agents are oxidized to hydrophilic sulfoxide groups, leading to the disintegration of clustered nanoparticles. This breakdown of the ^19^F MRI agents extends the T_2_ relaxation times, resulting in an increase in ^19^F NMR signal intensity. In Figure 7c,d, ^19^F MRI of different polymers in solution before and after oxidation demonstrated a significant response to ROS, transitioning from an ”OFF” to an ”ON” state. These novel polymeric ^19^F MRI agents hold promise for improved diagnosis and treatment of diseases characterized by high levels of ROS.

The extensive use of antibiotics, antivirals, and chemotherapy medications could potentially induce tissue inflammation and organ damage, elevating ROS and RNS levels in the body and further straining patients. Thus, the development of real-time and deep tissue imaging techniques for monitoring ROS and RNS levels in vivo is crucial and time-sensitive. Li et al. documented the creation of two responsive molecular probes for simultaneous visualization of •O_2_^−^ and ONOO^−^ in live subjects using ^19^F MRI [40]. These probes incorporate a signal modifier, a fluorine-containing segment with a distinct ^19^F chemical shift, and breakable connections that selectively respond to •O_2_^−^ and ONOO^−^, respectively. The presence of the signal modulator led to a notable decrease in relaxation signal for both probes, diminishing the ^19^F output signal. Once they could respond to •O_2_^−^ and ONOO^−^ appropriately, the linkers could be specifically cleaved, resulting in reduced paramagnetic relaxation enhancement effect, restoration of the ^19^F relaxation time, and emergence of a strong ^19^F MRI signal. Studies demonstrated the prompt and specific response of these probes to their target molecules.

To facilitate a direct comparison of representative fluorinated colloidal nanosystems for ^19^F MRI, key quantitative parameters such as detection limits and imaging conditions are summarized in Table 1. Despite the promising sensitivity and specificity of fluorinated colloidal nanosystems for ^19^F MRI applications, several technical and biological challenges remain to be addressed. Achieving sufficient MRI signal intensity often requires high fluorine loading, which can induce nanoparticle aggregation and compromise colloidal stability. Meanwhile, although these nanosystems have demonstrated favorable biocompatibility and multifunctionality in biomedical applications, their long-term biological safety remains insufficiently explored. In particular, systematic investigations into chronic toxicity, tissue accumulation, biodegradability, immune responses, as well as in vivo biodistribution and clearance across different organs are still limited. Therefore, future research should emphasize the rational design of fluorinated nanoplatforms with improved fluorine utilization efficiency and dispersion stability, coupled with standardized in vitro and in vivo toxicological evaluations, to facilitate their reliable clinical translation.

## 4. Surface Coating Agents

It is widely known that fluorinated polymers have attracted significant attention from hydrophobic surfaces that demonstrate strong water repellent properties because of their naturally low surface free energy. Additionally, their properties such as optical clarity, thermal resilience, and resistance to chemicals have led to these fluorinated polymers being applied in functional coatings for surfaces. Super-hydrophobic surfaces, inspired by naturally water-repellent surfaces like lotus leaves, rose petals, geckos, and springtails, have garnered immense interest from both scientific and industrial sectors. For instance, super-hydrophobic surfaces have water contact angles exceeding 150 degrees. Furthermore, super-amphiphobic coatings, which can repel water and organic liquids with low surface tension, exhibit significant contact angles above 150 degrees and minimal rolling angles below 10 degrees. These distinctive behaviors of super-wetting surfaces offer a multitude of potential applications such as self-cleaning, anti-fogging, antifouling, antibacterial properties, and oil–water separation, among others.

### 4.1. Self-Cleaning Application

**Figure 8:**
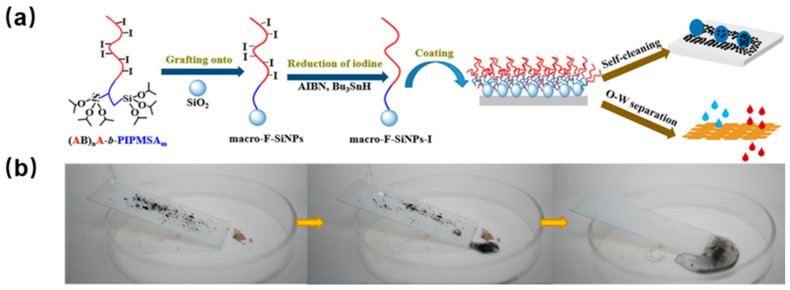
(**a**) Schematic representation of self-cleaning coatings using fluorinated silica nanoparticles. (**b**) Schematic diagram of self-cleaning application. Reprinted from Ref. [42] with permission from Elsevier.

The unique self-cleaning coating is created by establishing a special surface wettability on the material. Based on wettability principles, it is categorized into three types: super-hydrophobic, underwater super-oleophobic, and super-omniphobic coatings. For instance, Somdatta et al. developed FPB-g-SiNPs nanofillers by endowing two different types of fluorinated polymer onto the silica nanoparticle [41]. Then the FPB-g-SiNPs nanofillers can form multiscale roughness on the bare polystyrene surface, achieving excellent chemical durability, mechanical stability, and highly hydrophobic and self-cleaning ability. In a study, a fluorinated macromolecular coupling agent was synthesized using 1,6-diiodoperfluorane and α,ω-nonconjugated diene through step transfer-addition, radical-termination polymerization, and photocontrolled iodine-mediated reversible deactivation radical polymerization [42]. These agents were then applied to silica nanoparticles to boost the coating’s surface roughness. Moreover, the CF_3_ end groups of the fluoropolymers effectively lowered the solid surface’s free energy to achieve super-hydrophobicity (Figure 8a). As a result, the resultant coating exhibited stability against acidic, basic, and high-salt solutions. Meanwhile, the good super-hydrophobic performance of the coating was used for self-cleaning capabilities (Figure 8b).

### 4.2. Self-Healing Application

In recent years, self-repairing super-hydrophobic coatings have become a key focus in the field of multifunctional coating. The primary repair mechanism involves restoring the surface morphology and adding low-energy materials. Surface morphology can be recovered through dynamic chemical bonding and gas compensation, while the incorporation of low-energy substances depends on the migration of underlying species to the surface under specific conditions. Lin et al. successfully created a durable and super-amphiphobic fabric using a two-step wet-chemistry process with poly(vinylidene fluoride-co-hexafluoropropylene) polymer, fluoroalkyl silane, and modified silica nanoparticles [43]. The coated fabric showed exceptional resilience, enduring at least 600 cycles of standard laundry and 8000 cycles of abrasion without losing its super-amphiphobic properties. Furthermore, the coating displayed impressive resistance to exposure to strong acids/bases, ozone degradation, and boiling treatments. In the event of chemical damage, the coating could recover its original super-amphiphobic properties through a brief heating treatment or natural aging at room temperature. Han et al. developed a highly polymer-repellent fuoropolymer brush with excellent stability via surface-initiated atom transfer radical polymerization. The hydrophobic and antifouling characteristics of the surface could be easily restored multiple times through heating when damaged in acidic/basic conditions or UV exposure [44]. In addition, Chen et al. developed UV-responsive microcapsules loaded with fluoroalkyl silane through pickering emulsion polymerization with the assistance of titania (TiO_2_) and silica (SiO_2_) nanoparticles as the pickering agents [45]. These microcapsules were then sprayed onto a variety of substrates to synthesize a super-hydrophobic and self-repairing coating. Following mechanical damage or contamination with organic substances, the surface coating recovered its self-cleaning and super-hydrophobic properties under UV light, owing to release fluoroalkyl silane from the partially damaged capsules. The described functional coatings are environmentally friendly and particularly well-suited for outdoor use.

### 4.3. Antibacterial Application

**Figure 9:**
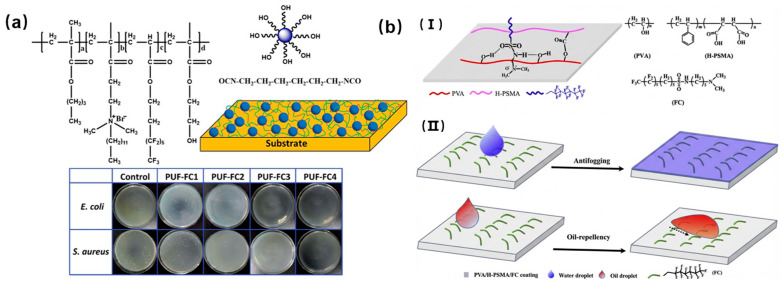
(**a**) The process of creating the PUF-FC coating and its effectiveness against bacteria. Reprinted from Ref. [46] with permission from American Chemical Society. (**b**) (**I**) Chemical structure and intermolecular interactions of PVA, H-PSMA, and FC. (**II**) Anti-fogging properties and oil-repellent mechanism of PVA/H-PSMA/FC coatings. Reprinted from Ref. [47] with permission from Royal Society of Chemistry.

The threat of bacterial contamination is a significant concern for human life and health. Hence, the development of versatile antibacterial materials is essential. One effective strategy to create such materials is the design of cationic polymers with antibacterial properties. These polymers are characterized by their cationic structures, including quaternary ammonium, phosphonium, pyridinium, and imidazolium groups. Since bacterial adhesion is the initial step in bacterial growth, an alternative approach is to create surfaces with low surface energy that inhibit bacterial adhesion, so fluoropolymers are commonly used for this purpose. For instance, Song and colleagues synthesized a copolymer consisting of poly(methyl methacrylate-co-ethyl acrylate-co-hexafluorobutyl methacrylate-co-isobornyl methacrylate) through free radical polymerization [46]. This copolymer contained borneol monomers and fluorine, imparting a unique crystalline cyclic alcohol structure with two enantiomeric forms. The complex composition and controlled release of borneol monomers disrupted the bacterial cell membrane, leading to antifouling and antibacterial properties. Additionally, the fluoropolymers provided the coating with low surface energy, making it difficult for bacterial adhesion. Consequently, the coating exhibited excellent environmental stability and versatility for various applications (Figure 9a). Zhang and colleagues conducted the synthesis of a copolymer containing fluorine and quaternary ammonium salt using free radical polymerization (Figure 9b), subsequently, the coating with a rough micro/nano structure was developed by linking the copolymer with hexamethylene diisocyanate and incorporating poly(ureaformaldehyde) particles [47]. The resulting composite coating displayed outstanding abilities for self-cleaning and repelling liquids, while keeping its super-hydrophobic nature even after exposure to 16 abrasion cycles and 20 cross-cut tape tests. Furthermore, the nanocomposite coating showed remarkable antibacterial properties against *Escherichia coli* and *Staphylococcus aureus* bacteria.

### 4.4. Anti-Fog Application

Fogging is a common issue that affects cold surfaces like windshields, glasses, and other transparent materials, leading to reduced optical transmission and performance. To combat this problem, researchers have developed functional coatings to prevent fog formation. Luo et al. developed a hydrophilic and oleophilic coating by incorporating a fluorocarbon surfactant into poly(vinyl alcohol) and hydrolyzed poly (styrene-co-maleic anhydride) (PVA/H-PSMA). Specifically, a fluorocarbon chain could make the PVA/H-PSMA surface amphiphilic and absorb water molecules, thus achieving anti-fogging properties. Additionally, the low surface energy of fluorinated alkyl chains enables oil droplets, which provide oil repellency. Li et al. created an anti-fogging and anti-icing coating by using polyhedral oligomeric silsesquioxane (POSS) along with poly[2-(dimethylamino)-ethyl methacrylate] (PDMAEMA) and poly(sulfobetaine methacrylate) (PSBMA). The hygroscopic nature of PDMAEMA and PSBMA segments of polymer networks can facilitate absorption of water or vapor molecules to enhance anti-fogging performance [48]. Meanwhile, a self-lubricating aqueous layer and hydrophobic POSS groups could decrease the water freezing point and fulfill the anti-icing property. In addition, Wang et al. developed a Y-shaped amphiphilic fluorinated monomer containing perfluoroalkyl and poly(ethylene glycol) side chains, which was then used to prepare a block copolymer with 2-(N,N-dimethylamino)ethyl methacrylate [49]. The block copolymers simultaneously exhibited excellent hydrophilicity and oleophobicity; thus, the resulting surfaces possessed unique anti-fog and oil-repellent properties.

### 4.5. Anti-Biofouling Application

**Figure 10:**
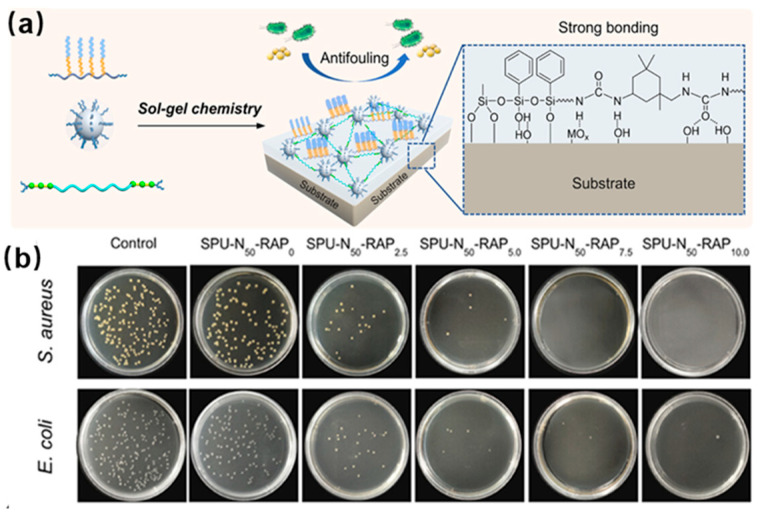
(**a**) Schematic diagram of polysiloxane antifouling coating. (**b**) Bacterial colony of polysiloxane coating with varying levels of active amphiphilic polymer. Reprinted from Ref. [51] with permission from American Chemical Society.

Biofouling is a common occurrence in both biomedical coatings and ship hull coatings, and it significantly hinders their long-term performance and practical application. Therefore, there has been a growing emphasis on combatting biofouling. Typically, biofouling surfaces rely on the molecular forces between external biomolecules and the artificial surface. Fluorinated polymers show low surface energy that can effectively diminish the interaction force, leading to antifouling properties. For instance, Ou and colleagues produced superb water-repellent chitin nanocrystal (ChNC) particles by combining tetraethyl orthosilicate and (3-mercaptopropyl) trimethoxysilane. Subsequent incorporation of sulfhydryl-functionalized ChNC particles with fluorinated long chains further decreased the surface free energy, resulting in a super-amphiphobic structure [50]. These super-amphiphobic nanoparticles were applied onto various surfaces, showcasing outstanding resistance to fouling and ease of cleaning due to their unique micro-nano architecture and low surface energy. As shown in Figure 10, Zhang et al. synthesized an antifouling and highly adhesive polysiloxane coating utilizing a bis-silane-terminated polyurea (SPU), an oligosiloxane nanocluster, and a bi-silanol-terminated poly(dimethylsiloxane) with fluorocarbon and poly(ethylene glycol) side chains as a reactive amphiphilic polymer (RAP) [51]. The amphiphilic segments of reduced surface energy could migrate to the surface, providing the coating with exceptional antifouling capabilities (Figure 10a). Simultaneously, the robust hydrogen bonds and silanes present could bind to the substrate, achieving strong adhesion for the coating. This coating demonstrated remarkable resistance to fouling from proteins and bacteria (Figure 10b), making it a suitable option for applications in medical devices and marine industries.

### 4.6. Oil-Water Separation Application

**Figure 11:**
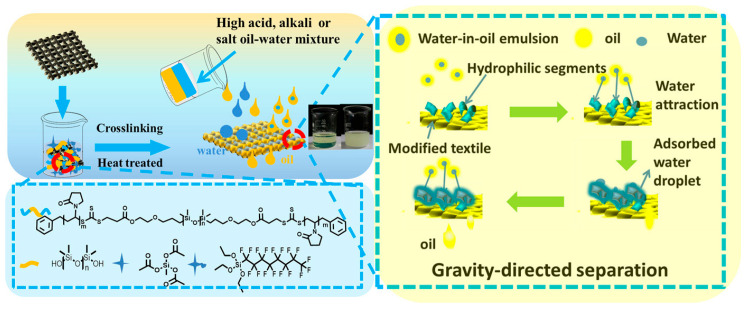
The schematic process of altering PDMS-FA-PVP coating on fabric and the separation process of emulsifying water-in-oil mixture. Reprinted from Ref. [53] with permission from Wiley.

Water pollution, specifically from oil, organic solvents, and dyes, has emerged as a significant environmental issue, posing threats to human health and the sustainable development of society. Due to the inherent immiscibility of water and oil, filtration materials with specific surface properties can be utilized to achieve efficient separation with minimal energy consumption. These materials allow for selective penetration of either water or oil upon contact. Recently, there has been a growing trend in using fluorinated polymers to create advanced filtration materials for oil–water separation, including hydrophobic–oleophilic and hydrophilic–oleophobic variants. Zhang et al. developed a high-flux oil–water separation material by applying PDMS-based fluorinated copolymers onto cotton textiles through a simple sol–gel method [52]. This innovative material not only enables the effective separation of oil–water mixtures with a flux reaching approximately 7500 L m^−2^ h^−1^ but also demonstrates efficacy in separating surfactant-stabilized water-in-oil emulsions. Remarkably, it maintains its performance even in harsh environments with elevated levels of acid, alkali, or salt. While hydrophobic–oleophilic materials repel the water phase and allow only oil to permeate, promoting efficient oil/water separation, they are susceptible to blockages or contamination by oil upon surface contact, leading to reduced pore penetration and diminished separation efficiency. Hydrophilic–oleophobic surfaces offer significant advantages over hydrophobic–oleophilic surfaces and are considered the ideal choice for oil/water separation due to their ability to repel oil. For instance, Zhao et al. developed a flexible super-oleophobic/super-hydrophilic surface, enabling precise control over oil transport and separation by combining fluorinated polymers with hydrophilic components and nanoparticles of varying sizes [53]. This innovative surface can be applied to diverse substrates to achieve regulated oil transport, efficient oil–water separation, and effective emulsion demulsification (Figure 11). By applying a super-oleophobic/super-hydrophilic coating to substrate surfaces, oil–water mixtures can be effectively separated. The achieved flux during the complete demulsification process reached an impressive 12,000 L m^−2^ h^−1^ bar^−1^.

Representative antifouling coatings based on fluorinated colloidal nanosystems and their key performance metrics, including water and oil contact angles, fouling species, and durability, are summarized in Table 2. Fluorinated colloidal nanosystems have demonstrated outstanding performance in antifouling, antibacterial, self-cleaning, anti-fogging, and oil–water separation applications, primarily owing to their low surface energy, selective wettability, and unique micro-/nano-structured surfaces. However, most of these systems are still confined to laboratory-scale demonstrations, and significant challenges remain regarding long-term durability, mechanical robustness, abrasion resistance, and stability under complex physiological or marine environments. In addition, issues such as membrane fouling, limited service lifetime, and scalability of fabrication processes hinder their practical implementation. Beyond performance-related limitations, increasing concerns have been raised about the environmental persistence, bioaccumulation, and long-range transport of fluorinated materials. Therefore, future research should not only focus on scalable and cost-effective fabrication strategies with validated performance under realistic service conditions but also emphasize the development of mechanically robust, environmentally benign, and partially degradable fluorinated or fluorine-reduced alternatives, supported by comprehensive life-cycle assessments and interdisciplinary collaboration.

## 5. Potential Toxicity and Environmental Impact of Fluorinated Colloidal Nanosystems

Despite the promising performance of fluorinated colloidal nanosystems in biomedical and functional coating applications, their potential toxicity to biological systems and the environment has attracted increasing attention. Previous studies have shown that certain fluorinated compounds, particularly long-chain per- and polyfluoroalkyl substances (PFAS), may exhibit bioaccumulation, long biological half-lives, and adverse effects on mammalian organs, including liver toxicity, endocrine disruption, and immunological effects. When engineered into nanoscale colloidal systems, additional safety concerns may arise due to their small size, high surface area, and enhanced cellular internalization, which can potentially induce oxidative stress, inflammatory responses, or membrane damage in vitro and in vivo. Furthermore, the environmental persistence of fluorinated materials poses potential ecological risks. Released fluorinated colloidal nanoparticles may accumulate in soil and aquatic environments during manufacturing, application, or degradation processes, where they can be taken up by microorganisms and transferred through the food chain. Although several studies have reported relatively low acute cytotoxicity for certain fluorinated nanomaterials at moderate concentrations, systematic long-term toxicological evaluations, including chronic exposure, biodistribution, metabolism, and clearance pathways, remain limited. To address these challenges, recent research efforts have focused on developing safer fluorinated nanosystems, such as short-chain fluorinated compounds, partially fluorinated or degradable polymers, and hybrid systems designed to reduce bioaccumulation and environmental persistence. In addition, comprehensive in vitro and in vivo toxicity assessments, along with standardized environmental risk evaluation protocols, are increasingly recognized as essential for the responsible development and practical application of fluorinated colloidal nanosystems. Further, interdisciplinary studies integrating materials science, toxicology, and environmental science are therefore necessary to fully elucidate their safety profiles and to guide future sustainable design strategies.

## 6. Perspectives and Future Challenges

Overall, fluorinated colloidal nanosystems have emerged as versatile and highly selective materials for biomedical and functional applications. Notable achievements have been made in ^19^F MRI, where these nanosystems enable high imaging specificity, low background interference, and potential quantitative detection. In addition, their favorable biocompatibility, structural tunability, and intrinsically low surface energy have enabled successful applications in biomedical platforms as well as antifouling, antibacterial, self-cleaning, anti-fogging, and oil–water separation coatings. Despite these advances, several challenges remain to be addressed. In biomedical and imaging applications, high fluorine loading required for sufficient ^19^F MRI sensitivity may induce nanoparticle aggregation and compromise colloidal stability, while long-term biological safety, including chronic toxicity, biodistribution, biodegradability, and immune responses, remains insufficiently understood. In functional coatings, issues related to complex fabrication processes, mechanical durability, scalability, and long-term stability under realistic service conditions continue to limit practical implementation. Moreover, increasing concerns regarding environmental persistence, bioaccumulation, and long-range transport of fluorinated materials must be carefully considered. Finally, future research should focus on the rational design of fluorinated colloidal nanosystems with improved fluorine utilization efficiency, enhanced dispersion stability, and comprehensive standardized in vitro and in vivo evaluations. At the same time, the development of mechanically robust, scalable, and environmentally benign fluorinated or fluorine-reduced alternatives, supported by life-cycle assessments and interdisciplinary collaboration, will be essential for enabling the sustainable and responsible translation of these materials into real-world applications.

## Figures and Tables

**Table 1:** Quantitative benchmarking of representative fluorinated colloidal nanosystems for ^19^F MRI and biological detection.

System Type	Target	Detection Limit	Detection Conditions	Evaluation Mode	Ref.
FCy7-NO_2_	Enzyme Detection	≤1.5 μg mL^−1^ and 10–50 μg mL^−1^	Fluorescence /MRI	in vivo	[20]
Fluorinated polymer	Protein Detection	~25 nmol/μg	14.1T MRI	in vivo	[25]
Fluorinated micelles	pH Detection	0.16 mg mL^−1^	MRI	in vitro	[28]
Perfluorocarbon	Ions Detection	500 nM	Multicolor imaging/MRI	in vitro	[31]
Iron fluoride vinyl polymer	ROS/Reducing Environment	/	MRI	in vivo	[34]

**Table 2:** Quantitative comparison of representative antifouling and functional surface coatings based on fluorinated colloidal nanosystems.

Coating System	Application	Water Contact Angle (°)	Fouling Species	Durability/Stability	Ref.
Fluorinated silica nanoparticle coating	Self-cleaning	172.6°	Dust	High stability against acidic, basic, and salty solutions	[42]
Fluorinated silica microcapsules coating	Self-cleaning/ Self-healing	152°	Oil	2160 h	[45]
Fluorinated polymer coating	Antibacterial	89°	Bacteria/algae	>27 days	[46]
Perfluorinated side chain polymer	Anti-fog/oil-repellent	70°	Oil/water	/	[49]
Fluorinated polysiloxane coating	Anti-biofouling	100°	Biofilms	/	[51]
Fluorinated membrane coating	Oil-water separation	160°	Oil/water	>750 cycles	[53]

## Data Availability

No new data were created or analyzed in this study.

## Abbreviations

The following abbreviations are used in this manuscript:

MRI	Magnetic resonance imaging
NMR	Nuclear magnetic resonance
ROS	Reactive oxygen species
CAs	Contrast agents
Lgmn	Legumain
PDMS	Polydimethylsiloxane
PDMS-FA-PVP	Polydimethylsiloxane–fluoroalkyl–Poly(vinylpyrrolidone)
